# Engaging the ‘Missing Men’ in the HIV Treatment Cascade: Creating a Tailored Intervention to Improve Men’s Uptake of HIV Care Services in Rural South Africa: A Study Protocol

**DOI:** 10.3390/ijerph18073709

**Published:** 2021-04-02

**Authors:** Oluwafemi Adeagbo, Kammila Naidoo

**Affiliations:** 1 Africa Health Research Institute, Somkhele 3935, South Africa; 2 Department of Sociology, Faculty of Humanities, University of Johannesburg, Johannesburg 2006, South Africa; kammilan@uj.ac.za; 3 Department of Health Promotion, Education & Behavior, Arnold School of Public Health, University of South Carolina, Columbia, SC 29208, USA; 4 South Carolina Smartstate Center for Health Care Quality, Arnold School of Public Health, University of South Carolina, Columbia, SC 29208, USA

**Keywords:** men, stigma, HIV care, candidacy framework, South Africa

## Abstract

Men, especially young men, have been consistently missing from the HIV care cascade, leading to poor health outcomes in men and ongoing transmission of HIV in young women in South Africa. Although these men may not be missing for the same reasons across the cascade and may need different interventions, early work has shown similar trends in men’s low uptake of HIV care services and suggested that the social costs of testing and accessing care are extremely high for men, particularly in South Africa. Interventions and data collection have hitherto, by and large, focused on men in relation to HIV prevention in women and have not approached the problem through the male lens. Using the participatory method, the overall aim of this study is to improve health outcomes in men and women through formative work to co-create male-specific interventions in an HIV-hyper endemic setting in rural KwaZulu-Natal, South Africa.

## 1. Introduction

Globally, South Africa (SA) is disproportionately affected by the HIV epidemic and is constantly striving to reach the ambitious UNAIDS 90-90-90 target amidst the continuous rise in new HIV infections complicated by HIV-related stigma (patient-level and health services), access barriers [1], and the recent coronavirus (COVID-19) pandemic that led to the temporary or permanent closure of some health facilities. The topic of ‘missing men and boys’ in the HIV treatment cascade came out strongly as a challenge to HIV prevention strategies during the UN 90-90-90 Target Workshop at the 21st International AIDS Conference held in July 2016, as well as the 2018 AIDS Conference held in Amsterdam, given the high mortality and morbidity in men and failure to control the epidemic. The 2017 UNAIDS blind spot report reiterates the challenges as well as the benefits of men’s utilization of HIV services [2]. The South African HIV Prevalence, Incidence, Behavior, and Communication Survey 2017 reveals that most men are not aware of their HIV status and some of those living with HIV are not on treatment and 40% (whether on treatment or not) are not virally suppressed—particularly those aged 25–44 years [3]. The report indicates that the HIV incidence rate in young males aged 15–24 years increased by 11% for the first time in South Africa, low condom usage and early sexual debut before age 15 years is increasing among young males while age-disparate sex with older men continues to increase amongst adolescent girls [3]. Since most HIV infections in South Africa are attributed to heterosexual sex, the increase in unprotected sex and young women’s age-disparate sex with older men could be major drivers of the epidemic.

Furthermore, recent studies conducted in our locality in rural KZN revealed that most men living with HIV do not know their HIV status and those with such knowledge are not virally suppressed due to both social and institutional barriers such as stigma, lack of male-friendly HIV services, masculinity and gender roles, privacy, clinic hours and long waiting time [1,4,5]. This explains the vicious cycle of HIV transmission and high incidence rate in the country, particularly in KZN, which needs urgent attention. To achieve zero new infections and zero AIDS-related deaths in our setting [6], there is a need to design an innovative intervention for men aiming at an early diagnosis and treatment pathway for those who are positive whilst those who are negative are encouraged to remain so to end the epidemic. Thus, the current study aims to co-create a tailored male-specific intervention using the participatory method to improve HIV testing and treatment uptake among men in rural KZN. A theory of change will also be developed to support a realist process evaluation of the intervention. Given that most interventions in our locality are not male-friendly, we hypothesize that the creation of a male-specific intervention will improve men’s health outcomes and will reduce the HIV incidence rate in young women. If the intervention is successfully designed and evaluated, it will not only benefit men but their sexual partners and family members.

## 2. Study Aims and Objectives

The overall aim of this study is to co-create (with men) male-specific interventions to improve men’s health outcomes in a rural HIV hyperendemic setting.

Specific objectives to achieve this aim include:

1. To synthesize the existing locally relevant data (mostly qualitative) on barriers and opportunities to engage men in health care through secondary analysis of extant data at an Africa Health Research Institute in KZN.
2. To explore and deepen our understanding of the emergent themes from a male perspective through follow-up in-depth interviews with men, particularly those who do not engage with the different steps of the HIV care and prevention cascade, e.g., HIV testing, linkage to treatment, and prevention.
3. To present the findings of the analysis to the research community, partners, and policy-makers.
4. To use the results of analyses from Aims 1 and 2 and feedback from Aim 3 to co-create a tailored intervention for men using the participatory method.

## 3. Research Question

The study is operationalized with the following research question:

Would a tailored male-specific intervention encourage men and boys to test for HIV, link them to care, and stay in care in a rural HIV hyperendemic area?

## 4. Theoretical Framework

The intervention development will be guided by an adapted candidacy framework to further explore the barriers and facilitators of HIV care services uptake among men. The candidacy framework was originally developed in the United Kingdom to examine people’s engagement with healthcare services [7]. It places value on an individual’s choices in accessing healthcare services [8]. It has been adopted and used in different settings including KZN to investigate how men and young people navigate HIV care services [1,9]. According to the framework, individuals need to identify that they are eligible for a healthcare service, and they need to make concerted efforts to navigate the processes and present themselves at healthcare facilities to assert their candidacy for that service. An individual’s eligibility is further adjudicated by healthcare providers who decide their suitability. Once access has been granted, individuals need to navigate some pathways to access care, which requires an active response from the users [1,10]. We adopted the candidacy framework to elucidate some of the barriers identified in previous studies in our locality [1,5,6,11,12] and guide the development of a male-tailored intervention that will ease the pathway to accessing and engaging with HIV testing, treatment, and prevention services.

## 5. Methods and Design

This study adopts qualitative research techniques. A participatory action research approach led by men to co-create a tailored intervention to improve men’s HIV testing uptake and linkage. The following subheadings highlight the method, procedures, study population, and design, among others.

### 5.1. Study Setting, Participant Recruitment, and Population

This study is being conducted in the Africa Health Research Institute’s (AHRI) longstanding demographic surveillance area in northern KwaZulu-Natal in the Hlabisa sub-district of uMkhanyakude district. The study area is predominantly rural, and poor compared with other parts of South Africa, with high HIV incidence and unemployment (over 85% of young adults aged 20 to 24 years are unemployed) and the local language is isiZulu [11]. The study population will include males (*n* = 30) aged >18 years living in the research areas in the Hlabisa sub-district. Purposive sampling will be employed when recruiting for workshops with in-depth interviews (IDI) of participants who meet the study criteria. Working with the AHRI Youth Engagement Officer and data management team, the study research assistants will recruit and invite participants (including interested IDI participants), particularly younger males living in the research areas (especially those who do not participate in research and/or engage in HIV care) to participate in the workshop to design an intervention that is suitable for them to access HIV care services.

### 5.2. Study Inclusion and Exclusion Criteria

The study inclusion and exclusion criteria are summarized in Table 1 below.

### 5.3. Study Design and Data Collection Method

This study adopts qualitative research techniques such as in-depth interviews (IDIs) and participatory action research approach led by men to co-create a tailored intervention to improve men’s HIV testing uptake and linkage. We are combining two data sources, (1) review of completed qualitative studies on barriers and facilitators of HIV care services uptake among men and women at AHRI which includes DREAMS (UKZN reference: BFC339/16), HITS (UKZN reference: BFC398/16), STAR (UKZN reference: BFC311/18) and mAfrica (UKZN reference: BE435/17) studies; (2) follow-up telephonic in-depth interviews (*n* = 10—we chose telephonic interviewing to minimize physical contact due to COVID-19 regulations) to further explore themes that arose in these parent studies through a male lens and to stimulate discussions around male-specific tailored intervention, and to better understand the barriers and facilitators of HIV testing and treatment amongst men in our settings; (3) conduct two participatory workshops with men (with a limited number of men in an auditorium that can contain at least 60 people as per ‘physical distancing’ rule) to design a tailored intervention for men; (4) conduct two feedback sessions (via Teams/Zoom with key stakeholders (e.g., Department of Health [DOH], Department of Social Development [DSD], Community Advisory Board [CAB], Youth Champions etc.) to discuss important findings on barriers impeding on men’s uptake of HIV care services as well as to receive their inputs to the intervention co-created with men to overcome those barriers.

Both participatory workshops and IDIs will be conducted in IsiZulu and will be audio-recorded. The interviews will be conducted telephonically by AHRI trained social science research assistants and will take approximately 60 min in length depending on the participant’s responses, and this will enable the researchers to understand, contextualize, and explore some of the issues around the study. The smaller number of IDI participants in the qualitative study is allowed since deeper meanings of concepts and thematic areas are explored. To limit disturbances and ensure privacy, the IDI will be conducted telephonically in a private call center at AHRI, and audio recorded with interviewees’ consents. Prior to the interview, participants will be encouraged to use pseudo names instead of their real names to ensure anonymity. Reflective observation notes written during interviews and workshops will also form part of the data during analysis. The choice of the qualitative method, such as the participatory method, for the proposed study will give us an in-depth understanding of the issue at hand and allows participants (young and older males) to co-create intervention to address the barriers discussed above. The participatory method entrenched in an interpretivist approach is gradually becoming popular among researchers, and its success in co-creating interventions with study participants about issues that affect them is well documented [13,14,15,16]. Being part of the solution to an issue that affects them will give participants a sense of ownership and responsibility to sustain the intervention.

### 5.4. Workshop Structure and Feedback Sessions

#### 5.4.1. Workshop 1 (Face-To-Face)

The main purpose of this workshop (facilitated by the PI and two research assistants) is to design and develop an intervention (including the theory of change) based on the findings of the secondary analysis conducted. The findings from previous studies will be presented to participants to engage with and share their experiences with the group while thinking about how some of the challenges can be addressed. Participants will be divided into smaller groups and each group will present their interventions before everything is put together to form a ‘holistic intervention’. Building on the ‘Candidacy Framework’ [7] that highlights some of the challenges, participants will co-create (with AHRI researchers) a tailored intervention that will encourage men to access HIV care services without the fear of stigmatization. Due to COVID-19, a group of participants (*n* = 10 × three groups) will work together at a time (each group will come in at a different time) in an auditorium that can contain at least 60 people while the ‘physical distancing rule’ and other precautionary measures are observed. Each group will come once for the intervention co-development. Coupled with the AHRI COVID-19 protocol including screening, temperature check, and attendance register, participants will be provided with face masks and individual hand sanitizers during the workshop. Workshop facilitators will also be provided with face masks and hand sanitizers.

#### 5.4.2. Feedback Session 1 (Virtual)

We aim to present the findings of the secondary analysis (coupled with the workshop participants’ experiences) and the intervention developed (including the theory of change) to the CAB, public engagement unit (PEU), AHRI researchers, clinical staff, local health officials, etc. for their views and input. This will encourage collaborative efforts from health services users and providers to co-create a sustainable intervention that will benefit all. Due to COVID-19, feedback session participants will be encouraged to join the meeting virtually (via Zooms or Teams).

#### 5.4.3. Workshop 2 (Face-To-Face)

We aim to present the feedback from key stakeholders (CAB, health providers, PEU, etc.) to the workshop participants for final iterations of the intervention as well as the theory of change. This will give us the platform to brainstorm and deliberate about our initial design and the iterative processes. Due to Covid-19, a group of participants (*n* = 8–10 × three groups) will work together at a time (each group will come in at a different time) in a seminar auditorium that can contain at least 60 people while the ‘physical distancing rule’ and other precautionary measures are observed. Each group will come once for the intervention co-development. Coupled with the AHRI COVID-19 protocol including screening, temperature check, and attendance register, participants will be provided with face masks and individual hand sanitizers during the workshop. Workshop facilitators will also be provided with face masks and hand sanitizers.

#### 5.4.4. Feedback Session 2 (Virtual)

This session will be in form of a seminar where the theory of change and the intervention will be presented to scientists, partners, AHRI staff, health providers, CAB members, etc. Due to COVID-19, feedback session participants will be encouraged to join the meeting virtually (via Zooms or Teams).

### 5.5. Data Management

Both the interview and workshop audio files will be stored on password-protected AHRI PCs and only authorized personnel will have access to them. It is important to mention that audio files will be used for the purpose of this study only. Once the audio files have been transcribed, translated and quality controlled, they will be destroyed. Qualitative data will be stored in the form of Word files which can be uploaded into NVivo—qualitative data management program. To reiterate, all study-specific data will be stored on a secured server at AHRI with controlled access [11].

### 5.6. Qualitative Data Analysis

Digitally recorded interviews conducted in IsiZulu will be transcribed and translated into English while important points from the workshops, interviews, and feedback sessions will be written down. Data from the interviews will be coded using NVivo software. The software will be used for the classification and coding of identified themes from the interview transcripts. The research team will review the identified themes and interview transcripts for inconsistencies and sufficient representation of participants’ comments. Evolving themes that address the key focus of the study will be explored and analyzed thematically following an interpretivist approach [16]. We would also adapt the ‘Candidacy Framework’ as our theoretical lens to engage with the data during analysis.

## 6. Ethics Approval, Confidentiality, and Informed Consent

This study was approved by the Research Ethics Committee at the University of Johannesburg (UJ) (Reference: REC-02-067-2020) and the University of KwaZulu-Natal (UKZN), South Africa (Reference: BREC/00001372/2020). The study was approved by the Community Advisory Board (CAB) before the study was approved by the institutional review boards. The research assistants have been provided with adequate training on research ethics such as confidentiality and voluntary participation. We will ensure anonymity and confidentiality at all levels of the research process, and our reports or presentations will not contain study participants’ identifying information. Permission to audiotape qualitative interviews and workshops will be sought from the participants and all data will be kept secure, either in a password-protected computer or locked filing cabinet. Audiotapes and transcripts will be destroyed as soon as the analysis has been completed. Pseudo names will be used when reporting the qualitative data. Participants will be provided with adequate information about the study, and they will be allowed to ask questions for clarification prior to their involvement in the study. Voluntary informed consent will be ensured once participants have a full understanding of the study procedures. This study conforms to the ethical guidelines and standards of AHRI, UKZN, and UJ.

## 7. Dissemination Plan

The results of the study will be disseminated through peer-reviewed journal publications, local and international conferences, or symposia. Particularly, the findings of this study will be documented in form of success stories, reports as well as articles in accredited peer-reviewed journals. The study report will be shared with stakeholders from NDoH, AHRI, CAB, and other partners.

## 8. Discussion

Several studies have shown that men are less likely to test for HIV and link to HIV treatment if found to be positive, resulting in poor health outcomes such as unsuppressed viral load and higher AIDS-related death among men [5,6,12,17]. The recent Test-as-Prevention (TasP) trial conducted in rural South Africa had no significant effect [17]. One of the key recurrent issues from the TasP trial and other studies was that men and young people were not engaging with HIV care services due to several barriers such as stigma, clinic hours, unfriendly clinical services, and transportation [1,5,6]. Men (especially young men) have been consistently missing from the cascade of HIV care, leading to poor health outcomes in men and ongoing transmission of HIV in young women. Although these men may not be missing for the same reasons across the cascade and may need different interventions, early work has shown similar trends in men’s low uptake of HIV care services and suggested that the social costs of testing and accessing care are extremely high for men, particularly in South Africa [3,5,6,12,17,18]. Interventions and data collection have hitherto, by and large, focused on men in relation to HIV prevention in women and have not approached the problem through the male lens. Innovative male-tailored interventions that will improve HIV testing, treatment, and prevention uptake among men are needed to reduce HIV transmission and improve individual’s health outcomes.

A major strength of this study is the potential users’ involvement in the co-development of a theoretically-driven intervention to improve men’s engagement and utilization of HIV care services. A recent study conducted in KZN shows that men’s involvement in the development of an app increased their intrinsic motivation to test for HIV [6], while a financial incentive increased HIV testing uptake among men by more than 50% [5]. The use of a participatory research method and in-depth interviews in this study avail us the opportunity to engage men directly about issues that affect them and to come up with potential solutions to those issues. This will bring about a research partnership and a sense of belonging to the men as they will be developing an intervention that is suitable for their needs. If the intervention is successfully developed and most men know their HIV status (including linkage to antiretroviral treatment and pre-exposure prophylaxis), this will not only benefit men but also their sexual partners thereby reducing onward HIV transmission. Following the UNAIDS 2017 report on “missing men” and other calls not to leave males behind in HIV care, the findings of this study will address critical gaps and contribute to the literature on HIV testing, treatment, and prevention interventions for men in South Africa. Although the findings of this study may not be applicable in other provinces or regions in South Africa, it will lay the groundwork for future interventions to improve men’s utilizations of HIV care services without fear of stigmatization or perceived conflict with their masculinity.

## 9. Conclusions

The gains of large HIV testing, treatment, and prevention programs in South Africa will dwindle if men are left behind in HIV services given that most HIV infections in SA are contracted through heterosexual sex. Improving male’s access and uptake of HIV care services is key to South Africa’s efforts to eliminate AIDS by 2030. Therefore, the findings of this study will fill a critical gap in knowledge and show how men can be effectively engaged in HIV care in a resource-constrained setting.

## Figures and Tables

**Table 1:** Study inclusion and exclusion criteria.

Inclusion Criteria	Exclusion Criteria
Participants must be male aged 18 years and older	Anyone less than 18 years
Participant must consent to participate in the study	Anyone who identifies as a female
Participants must be a resident within the AHRI research areas	Anyone who is unwilling to consent

## Data Availability

Although we did not report any data in this manuscript, the current study data will be made available upon written request when the fieldwork and data processing have been completed.
